# Identification of Requirements for Computer-Supported Matching of Food Consumption Data with Food Composition Data

**DOI:** 10.3390/nu10040433

**Published:** 2018-03-30

**Authors:** Barbara Koroušić Seljak, Peter Korošec, Tome Eftimov, Marga Ocke, Jan van der Laan, Mark Roe, Rachel Berry, Sandra Patricia Crispim, Aida Turrini, Carolin Krems, Nadia Slimani, Paul Finglas

**Affiliations:** 1 Computer Systems Department, Jožef Stefan Institute, Ljubljana 1000, Slovenia; peter.korosec@ijs.si (P.K.); tome.eftimov@ijs.si (T.E.); 2 National Institute for Public Health and the Environment (RIVM), Bilthoven 3720, The Netherlands; marga.ocke@rivm.nl (M.O.); jan.van.der.laan@rivm.nl (J.v.d.L.); 3 Quadram Institute Bioscience, Norwich, Norfolk, NR4 7UA, UK; mark.roe@quadram.ac.uk (M.R.); Rachel.Berry@quadram.ac.uk (R.B.); paul.finglas@quadram.ac.uk (P.F.); 4 International Agency for Research on Cancer (IARC), Lyon 69008, France; sandracrispim@gmail.com (S.P.C.); slimanin@iarc.fr (N.S.); 5 Department of Nutrition, Federal University of Paraná, Curitiba 80210-170, Brazil; 6 CREA—Council for Agricultural Research and Economics, Research Center Food and Nutrition (CREA-AN), Rome 00198, Italy; aida.turrini@crea.gov.it; 7 Max Rubner-Institut, Karlsruhe 76131, Germany; carolin.krems@mri.bund.de

**Keywords:** food matching, food composition, food consumption, optimization algorithm

## Abstract

This paper identifies the requirements for computer-supported food matching, in order to address not only national and European but also international current related needs and represents an integrated research contribution of the FP7 EuroDISH project. The available classification and coding systems and the specific problems of food matching are summarized and a new concept for food matching based on optimization methods and machine-based learning is proposed. To illustrate and test this concept, a study has been conducted in four European countries (i.e., Germany, The Netherlands, Italy and the UK) using different classification and coding systems. This real case study enabled us to evaluate the new food matching concept and provide further recommendations for future work. In the first stage of the study, we prepared subsets of food consumption data described and classified using different systems, that had already been manually matched with national food composition data. Once the food matching algorithm was trained using this data, testing was performed on another subset of food consumption data. Experts from different countries validated food matching between consumption and composition data by selecting best matches from the options given by the matching algorithm without seeing the result of the previously made manual match. The evaluation of study results stressed the importance of the role and quality of the food composition database as compared to the selected classification and/or coding systems and the need to continue compiling national food composition data as eating habits and national dishes still vary between countries. Although some countries managed to collect extensive sets of food consumption data, these cannot be easily matched with food composition data if either food consumption or food composition data are not properly classified and described using any classification and coding systems. The study also showed that the level of human expertise played an important role, at least in the training stage. Both sets of data require continuous development to improve their quality in dietary assessment.

## 1. Introduction

Constant changes in the food supply and modifications of our eating habits necessitate collecting food consumption data as well as evaluating food composition data over time. Food consumption data are prerequisite for evaluating inadequacies in dietary intake of key nutrients and risks related to possible hazards in food, for allowing estimates of consumers’ exposure to such hazards by linking to chemical occurrence data [1]. Food composition data carry information about nutritionally relevant components of foods, and provide values for energy and nutrients including macronutrients, vitamins and minerals and other important food constituents [2,3]. Both types of food-related data are important in many fields including clinical practice, research, nutrition policy, public health and education, and the food manufacturing industry [4]. To enable the application of food consumption and composition data, countries have gathered food databases where data is described, aggregated and ordered using comprehensive food description and classification systems [5]. However, as food consumption and composition databases describe and classify (and/or aggregate) data in different ways, it makes food matching between the two databases a difficult task, as also illustrated in the FAO (Food and Agriculture Organization of the United Nations) guidelines for food matching [6].

In the 1980s, the EU (European Union) started to support coordinated actions aimed at overcoming the lack of internationally comparable data [7], both in food consumption and food composition data. The FLAIR-Eurofoods-Enfant- [8] and the COST99-coordinated [9] actions dealt with both these subjects. Subsequently, the DG-SANCO (Directorate General for Health and Consumer Affairs) of the European Commission supported the DAFNE (Data Food Networking) initiative, which concerned the creation of a European food consumption dataset based on Household Budget Surveys [10]. Today, there are two strong umbrella organizations that unify work on food consumption and food composition data in Europe, i.e., the European Food Safety Authority (EFSA) and the international non-profit association EuroFIR [11]. EFSA gathers food consumption data for individuals in Europe and uses them for exposure assessment. Food consumption data collected from EU member states are stored in the Comprehensive Food Consumption Database, using a common food classification and coding system for classifying and describing food that makes it easier to compare data from different sources and perform more detailed types of data analysis FoodEx (for more details please refer to Section 2.1.1) [12]. The fundamental aim of EuroFIR has been the harmonisation and the standardisation of the work on food composition data in Europe. It has supported the development of the European standard for food composition data [13], which facilitates access to and exchange of comparable, high quality food composition data for industry, regulators and researchers across Europe [14,15]. As for the food classification and coding system, the EuroFIR member countries use the LanguaL system that is a globally defined automated method for describing, capturing and retrieving food information (for more details please refer to Section 2.1.2), with the exception of the German Nutrient Database [16]; and since 2012, some member countries have adopted the FoodEx system as well.

To link food consumption with food composition data, food matching is required to identify the most appropriate foods in the most appropriate compositional data sources. The International Network of Food Data Systems (FAO/INFOODS) prepared guidelines for food matching, where the needs and challenges of food matching are well described [6].

The aim of this paper is to identify the requirements for computer-supported food matching, in order to address not only national and European but also international current needs. This is as integrated research contribution of the FP7 EuroDISH project [17,18].

A new concept for food matching based on optimization methods is proposed, after having briefly described the available classification and coding systems and the specific problems of food matching. To illustrate and test this concept, a study was conducted in four European countries using different classification and coding systems. This real case study enabled us to evaluate the new food matching method and to provide further recommendations for future work.

## 2. Materials and Methods

### 2.1. Systems for Describing Food Information

EFSA recommends that food consumption data across Europe are collected by using the dietary record method for infants and children and the 24-h recall method for adults [1]. These methods use open-ended food sections, i.e., the food list is not a priori defined, in order to capture the most detailed food description so allowing for matching consumption data with food composition data [19,20]. This is usually performed by using software that collects food consumption data in a structured way asking supplementary information on foods and practices (e.g., brand name, packaging method, cooking procedures and other specific information, such as fortification). Collected data can then be classified (aggregated) in FoodEx groups using the core food list (level 4 and above) and coded using FoodEx facets and facet descriptors that are terms representing supplementary information describing food production and processing. It is also recommended by EFSA [1] that a food propensity questionnaire should be used to collect information on frequency of food consumption, particularly those consumed infrequently, and to correct for within-subject variability of food records or recalls.

Countries use different software for collecting food consumption data. An example of such a data processing software is GloboDiet (initially known as Epic-Soft), which is a standardised interview-based dietary assessment (24 h-recall) tool for obtaining a very detailed and comparable description and quantification of foods, recipes, and supplements consumed in the course of the preceding day across countries. It was initially developed within the EPIC study (European Prospective Investigation into Cancer and Nutrition) by the International Agency for Research on Cancer (IARC) in collaboration with national EPIC centers [21]. More recently, it has been adapted for European nutritional surveillance [22,23,24] or other national contexts outside Europe [25,26]. EFSA has also recently evaluated a sample of data processing software that can be used alone or in a combination with GloboDiet [27].

Food consumption data and occurrence data are usually merged in the context of a probabilistic assessment of dietary exposure (for example, see the Monte Carlo Risk Assessment software [28], developed in the context of the European project “Montecarlo” [29]), while for energy and nutrient intake calculations the linkage between food consumption data and food composition tables has a deterministic characterization [30]. Once food consumption data are collected and classified, the matching of consumed foods with the most appropriate sources of compositional data needs to be performed. This is a time-consuming procedure that requires skilled persons with expert knowledge of food composition and nutrition. Often foods are ambiguously described with insufficient detail to enable exact matches to food composition data, which makes the task even more difficult.

How food consumption data considered in this study was collected is described in more details in Table 1.

#### 2.1.1. The FoodEx System

**Figure 1:**
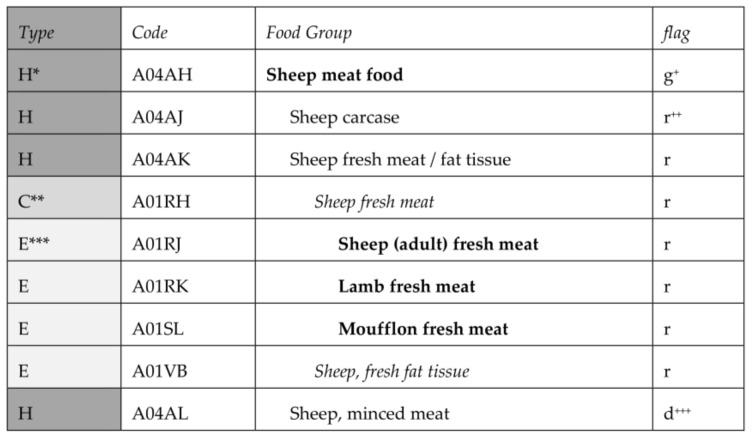
Example of FoodEx2 description and classification.* H—‘hierarchy term’; ** C—‘core term’; *** E—‘extended term’; ^+^ g—‘group’; ^++^ r—‘raw’; ^+++^ d—‘derivative’.

FoodEx stands for “Food classification and description for Exposure assessment” [1]. The current FoodEx2 version consists of a large number of individual food items aggregated into food groups and broader food categories in a hierarchical structure of parent-child relationships as nest hierarchy as there are multiple levels included (Figure 1). Central to the system is a core list of 1164 food groups or individual food items that represents the minimum level of detail needed when coding or identifying a food collected in any domain for intake or exposure assessments. More detailed terms may exist below the core list and these are identified as the extended list (1509 terms). Apart from bearing a unique alphanumerical code, all terms in FoodEx2 are flagged with attributes defining their role (hierarchy, core list or extended list) and their state (e.g., raw commodity, ingredient, simple or composite food).

#### 2.1.2. The LanguaL System

LanguaL stands for “LanguaaLimentaria” or “language of food”. It is an automated method for describing, capturing and retrieving food information. In LanguaL, each food is described by a set of standard descriptors (indexing terms) chosen from the following facets of the nutritional or/and hygienic quality of the food: A (Product Type), B (Food Source), C (Part of Plant or Animal), E (Physical State, Shape or Form), and F to Z (additional descriptors for indexing the product information). The LanguaL facet terms are fully structured in a hierarchy, which enables displaying its thesaurus in a logical way. Each term may have several narrower terms giving the concept a more specific meaning. The hierarchy also possesses poly-hierarchical relationships, meaning that a term may be related to several broader terms representing the concept in a wider meaning.

**Figure 2:**
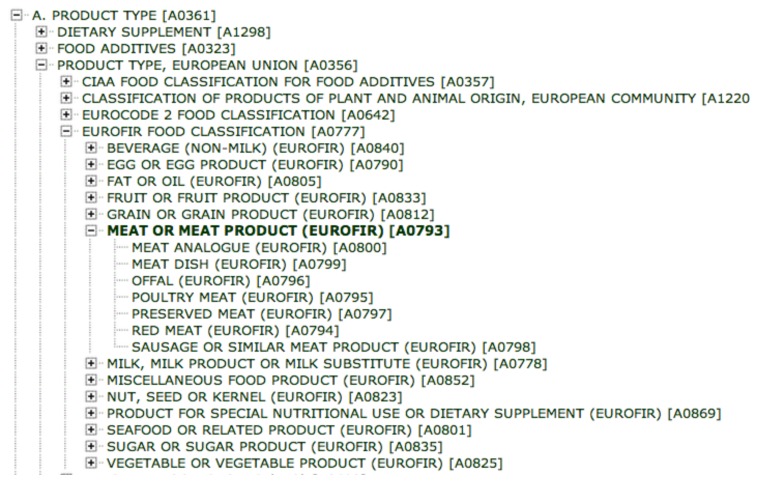
Example of LanguaL description and classification.

Each food can be allocated to several food group classifications (Facet A Product type—Figure 2). At the moment, LanguaL includes 13 classification systems, including Codex Alimentarius [31], European Food Groups, EuroFIR Food Group Classification, GS1 (Global Language of Business) Global Product Classification [32] and USDA (U.S. Department of Agriculture) standard reference [33]. The GloboDiet food groups, used to ease data collection and standardisation across countries, are currently not included. Over 75,000 foods and food products have already been indexed in various countries using the LanguaL system. Further details can be found in the LanguaL thesaurus [34].

### 2.2. Formalisation of the Food Matching Problem

There are two major challenges with food matching. Firstly, not all countries have compiled food composition databases of the same quality and national food composition databases may include different numbers of food items and data for nutrients. Secondly, food composition databases vary in the number of food items that have already been indexed by the available classification and coding systems. Different levels of detail, in which food items are described, can translate into lack of information and matching to incorrect or sub-optimal composition data, e.g., “bread” is a generic description that may be sufficient in some cases but does not allow identification of additional descriptors that may influence nutritional content, e.g., white/brown/wholemeal, with/without added ingredients. 

To formalize food matching as a problem to be solved in an algorithmic way, we defined food data in the following way:

- A *food consumption datum* is described by the English name, food group and subgroups, and a list of facets and descriptors, belonging either to FoodEx or any other (e.g., GloboDiet) food consumption classification (aggregation) and coding system;
- A *food composition datum* is described by the English name and a list of LanguaL facet terms. All Facet A “named Product Type” terms are associated to food groups and subgroups of a certain classification system (e.g., Product type European Union includes CIAA (Confederation of the Food and Drink Industry of the EU) Food Classification for Food Additives; Classification of Products of Plant and Animal Origin, European Community; Eurocode 2 Food Classification; Eurofir Food Classification; European Food Groups (EFG); Product Type, International).

Unfortunately, in many cases datasets do not include all the necessary information, which makes searching for food matches inherently less accurate. However, searching can be performed even when not all the information is provided.

During the food matching process, an optimal food composition data match needs to be found for each food consumption data. The matching can be done by the English names, food groups and subgroups, and the lists of FoodEx and LanguaL descriptors. If there is no food composition data that fully matches with food consumption data, a set of closely matched but suboptimal food composition data should be provided to be explored by a human expert.

### 2.3. Optimization Algorithm for Food Matching

There are many optimization algorithms that could be used for matching foods described in different ways. In this study, we focused on combinatorial optimization (a domain in applied mathematics and theoretical computer science), which consists of finding an optimal solution (in our case, the best match between selected food consumption and/or composition items) from a finite set of possible solutions. This set is defined by gathering an existing knowledge about food classifiers, as well as about food facets and terms. We created the initial set of optimal solutions (i.e., trained the algorithm) using the knowledge obtained from human experts, who were experienced experts in the field. Few of the experts involved were also the authors of this manuscript, i.e., M.O. from The Netherlands, C.K. from Germany, and R.B. from the UK. Through the iterative usage of the algorithm, this knowledge expands and the algorithm becomes more and more efficient in finding optimal solutions.

The following pseudo-code presents an idea of computer-supported food matching:

**Step 1:**  The human expert provides an input query that classifies a food item from a Food Consumption Database by its English name, food group and subgroups, and describes it by a set of facets and descriptors (e.g., FoodEx facets and descriptors);
**Step 2:**  The algorithm searches for the best matched data from Food Composition Database using food composition descriptors (e.g., LanguaL descriptors);
**Step 3:**  Acquired matches are displayed—perfect matches, which have all components of the input query the same, are displayed at level 0; while less perfect matches are displayed at the next levels, where matches at level 1 are food items that match with the query in all attribute groups except in one, matches at level 2 are food items that match with the query in all attribute groups except in two, etc.;
**Step 4:**  The human expert either selects the best match or amends the input query;
**Step 5:**  The information about the matched food consumption and food composition data is stored for algorithm training (learning) purposes;
**Step 6:**  Repeated from step 1 until all foods are matched.

### 2.4. Real Case Study Design to Test the New Food Matching Concept

In the study, we performed three kinds of food matching that could be combined.

- In the first case, we developed an algorithm to support matching food consumption data classified (aggregated) by GloboDiet with food composition data described by LanguaL. The GloboDiet classification system differs from FoodEx in both facets and groups/subgroups;
- In the second case, we extended the algorithm to support matching food consumption data classified (aggregated) by FoodEx with food composition data described by LanguaL;
- In the third case, the algorithm was adapted for matching between food composition data described by LanguaL.

This kind of matching is useful when one food composition database from a selected country needs to be matched with another food composition database from another country and the matching cannot rely only on English names because these are missing or are improperly selected.

In addition to the algorithm, we developed a simple web-based application, where information about food consumption data is provided as an input query, and after running the algorithm, matched food composition data are displayed. As the aim of the study was to identify requirements for efficient food matching, this application was developed for testing purposes only. It was used by human experts to evaluate food matches.

In the following subsections, three different types for matching foods described using different systems (GloboDiet, LanguaL and FoodEx) are provided. In all approaches, the human expert provides an input query that classifies food consumption data with food composition data, which is needed to estimate nutrient intake from food consumption. The algorithm explores the space of optimal solutions and outputs either a perfect food composition data match or a set of candidates, which are described by their codes, English names, basic nutrient values, and facet terms. The best matches at level 0 are food items from the food composition dataset that match with the query in all attribute groups (i.e., in at least one food name, the group, and all the facets and facet descriptors). If the query does not include the food names, these are not considered for matching and level 0 means matching in all other attribute groups (i.e., in the group, and all the facets and facet descriptors). The same logic applies if some other attribute group is missing in the query. Matches at level 1 are food items that match with the query in all attribute groups except in one, matches at level 2 are food items that match with the query in all attribute groups except in two, etc. The number of levels is unlimited and may vary from case to case depending on the number of available attributes in the input query. It is the responsibility of the human expert to decide on the best solution, to define the overall quality of the estimation of the nutrient profile and correct the search attributes if needed. The information is stored for further searches.

#### 2.4.1. Matching Food Consumption Data Described by GloboDiet to Food Composition Data Described by LanguaL

In this approach, each food consumption data is described by its English name, food group, food subgroups, and/or a set of GloboDiet facets and facet descriptors. Using the food matching algorithm, this information needs to be provided in a form of an input query. An example of such a query is “XCourgette, Xraw, G02, 0499, 0204, 0309”, where the first two terms present the English name of the food item from the food consumption database (Courgette, raw), G02 is the GloboDiet group code 02 (Vegetables), and 0499, 0204, 0309 are facets and facet descriptors (Preservation method—fresh, Physical state/form—flesh and Cooking method—fatty—stir fried/sautéed). The symbol “X” annotates the terms describing the food name (“XCourgette” and “Xraw”).

**Figure 3:**
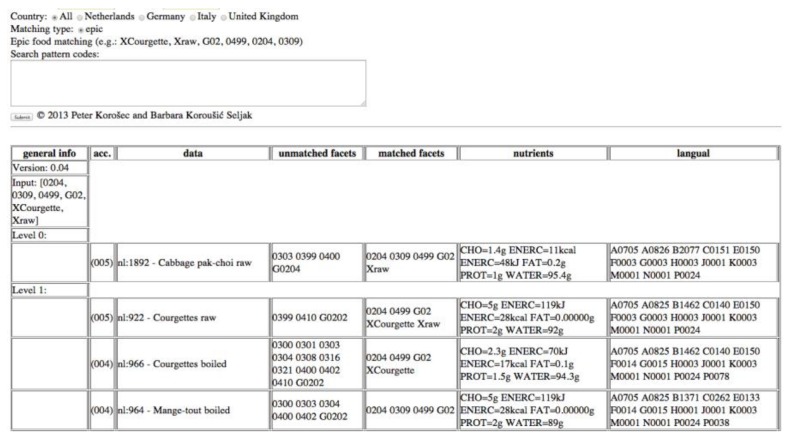
GloboDiet to LanguaL food matches.

In Figure 3, we present how the algorithm provides matches for the input query. In this approach, facet terms are LanguaL terms. The nutrient values include the values of the following EuroFIR components: carbohydrates (CHO), calculated energy (ENERC), total fat (FAT), protein (PROT) and water (WATER). The information about the matched and unmatched facets is also displayed alongside these data. Figure 3 shows an example, where the best match is the food named “Cabbage pak-choi, raw” because the query was specified with the names XCourgette and Xraw, the group G02, and the descriptors 0499, 0204, 0309. The food named “Cabbage pak-choi, raw” matches with the query in at least one name (“raw”), the group G02, and the descriptors 0499, 0204, 0309. The food named “Courgettes, raw” is specified as a level 1 match because it does not match with the query in the facet group (it is missing the facet descriptor 0309, which describes the cooking method: Fatty, stir fried/sautéed).

#### 2.4.2. Matching Food Consumption Data Described by FoodEx2 to Food Composition Data Described by LanguaL

The human expert provides an input query that classifies food consumption data by its English name, food group and subgroups, and describes it by a set of facets and facet descriptors. An example of such a query is “Xmargarine, A039D, F10.A0B8M”, where the first term presents the English name of the food item out of the food consumption database (Margarine), A039D is the FoodEx term (Traditional margarine), and F10.A0B8M is the FoodEx facet descriptor (Low sodium/reduced salt) from the FoodEx facet F.10 (Qualitative-info facet). The symbol “X” annotates the term describing the food name (“Xmargarine”).

**Figure 4:**
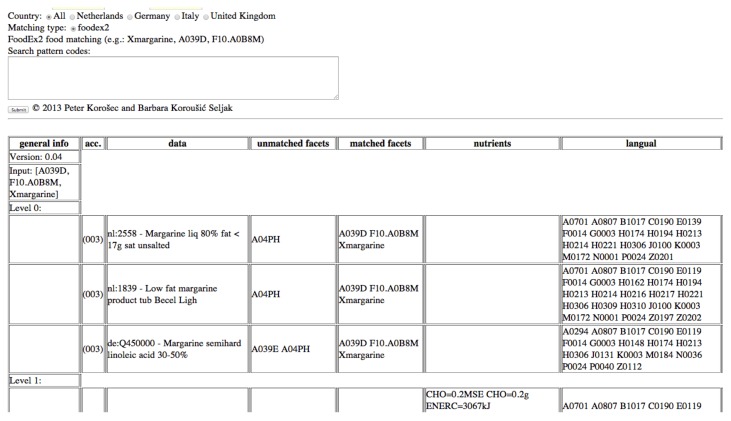
FoodEx2 to LanguaL food matches.

In Figure 4, it can be seen that three food items were identified as best matches because all of them match with the name (Margarine), the term A039D, and the facet F10.A0B8M.

#### 2.4.3. Matching between Food Composition Data Described by LanguaL

If a solution for food consumption to food composition matching misses the nutritional values of the compositional data, the algorithm further searches for the best matches in food composition data considering the LanguaL facet terms. The approach takes advantage of gathering new knowledge from human experts using the system, as well as complex information from various food datasets.

## 3. Evaluation

The food matching approach was evaluated together with experts from Germany, The Netherlands, Italy and the UK, under the the FP7 EuroDISH project framework.

### 3.1. Training Stage

In the first stage of the evaluation, the EuroDISH partners from Germany, The Netherlands and Italy prepared subsets of food consumption data that had already been manually matched with national food composition data, for training purposes. UK data were not used for training purposes as the national dietary survey in the UK uses manually recorded food diaries (i.e., paper diaries), which are then reviewed and coded to food composition data by trained experts. The resultant food composition codes are then entered in to the dietary analysis software, and therefore, no electronic datasets are available that include both the food consumption information (as recorded by the subject) and the food composition data. In Table 1, details about the training datasets from all three countries are provided. These datasets were prepared considering data from national food consumption surveys. While the German and the Dutch food consumption data were collected using the GloboDiet system, the Italian data were collected by another system (INRAN-DIARIO).

Subsets of food composition data for The Netherlands, Italy and the UK were selected from the EuroFIR Information Platform that had already been indexed by LanguaL. As the German Nutrient Database (BLS) was not available in the EuroFIR Platform, a subset of about 1200 of the 14,800 BLS food items coded by LanguaL was prepared for the case study. Details of the training food composition data subsets are presented in Table 2.

To enrich the training datasets, we applied FoodEx2 indices of food composition prepared by EuroFIR for EFSA in the project “Updated food composition database for nutrient intake”. There were also few FoodEx2 facet descriptors from several facet groups that EFSA has changed since the project Also these descriptors were excluded from the training datasets.

### 3.2. Testing Stage

After the training stage, the food matching approach was tested on food consumption data prepared by Germany, The Netherlands, Italy and the UK. Firstly, 40–50 foods were selected for each country. Details about the foods for Germany, other than the survey data used in the training dataset, were reported in a recent food consumption survey [40]. Also for The Netherlands the selected foods were taken from another survey [41] than the training dataset. For Germany, The Netherlands and Italy, the selected foods had already been manually matched with food composition data. For the UK, food items were not derived from actual food consumption data as this would have required accessing original, hard copy subject diaries from the National Diet and Nutrition Survey. Instead, a list of foods was drawn up to fulfil the testing exercises categories, and described in the level of detail typically found in volunteer diaries. The list was formulated by experts at the Quadram Institute Bioscience who were familiar with the UK food composition dataset and knew where particular matching difficulties could be encountered. A number of specific aspects were considered in the selection of the foods (some were not feasible for all countries) in order to test a wide variation of foods. The aspects covered included:

- Foods that can be best matched (i.e., known from previously matched databases)
- Foods that can be matched to a sufficient standard (as judged by the human expert)
- Foods for which no acceptable item in the food composition data was present
- Foods that are primary agricultural products
- Foods that are processed in various ways, including prepared with fat
- Complex commercially prepared foods with many ingredients
- Homemade recipes
- Branded foods
- Fortified foods
- Generic or non-specified foods
- Foods that can be matched to FoodEx classification at different levels (only tested for Italy)
- Foods from different FoodEx food groups (only tested for Italy)

Human experts from Germany and The Netherlands performed validation of the GloboDiet to LanguaL food matching approach [42], while human experts from Italy and the UK validated the FoodEx to LanguaL food matching approach [43]. The matching exercise in the UK was completed using food names only (without facets or descriptors) because only information at the subject-diary level was available.

The German, Dutch and Italian experts validated food matching by selecting best matches from the options given by the matching algorithm without seeing the result of the previously made manual match. The expert in each country completed an evaluation form with the matching results. The form recorded the number and type of foods from the consumption survey for which a matching food was found by the food matching algorithm. In addition, the quality of the selected best match (good similarity, sufficient similarity or poor similarity) and the reason for selecting a match from different options was indicated. The foods selected as best matches were compared with the matches selected previously using the existing country-specific ‘manual matching procedures’. In the UK, the matching was undertaken by two researchers of varying experience to test some of the potential limitations that the algorithm may have, particularly as the matching within the UK is based purely on text terms (no facet codes). The results of each match were compiled into one spreadsheet, and both matches were compared to the expected match. The expected match was based on independent agreement of the best available match in the dataset, i.e., the match or course of action that would be taken if the foods were coded as part of a current intake study. Since only level 0 (best quality) matches were obtained, these were categorised on the number of matching words.

Finally, in all countries an additional round of matching was conducted including composition datasets from other countries to determine whether any improved matches could be achieved using the additional composition datasets (number of matches improved compared to national dataset match).

## 4. Results

**Figure 5:**
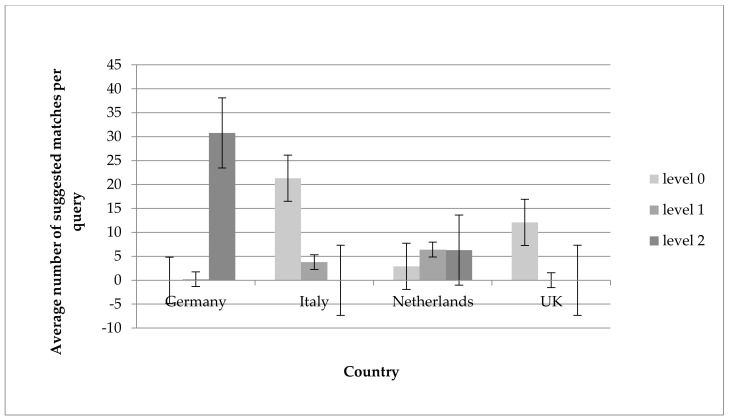
Estimated average number of suggested matches per query by quality level (level 0 is best quality) as indicated by the matching tool. Matches of level higher than 2 are not presented in the graph.

In all countries, the matching algorithm provided matches for all foods that were given as input for the matching algorithm. Figure 5 shows the estimated average number of possible matches for quality level 0 (best quality), quality level 1 (one aspect not matched), and quality level 2 (two aspects not matched) in each country, as provided by the algorithm. The provided averages are only estimates since in cases where the algorithm provided many possible matches, only a crude classification of the number was given (e.g., >50). For the four countries, varying average numbers of matches and different divisions over the three quality levels were observed. Of the 45 foods from the German dataset, 1 food (2%) matched at level 0, and 31 foods (69%) matched at level 2. In the Italian dataset, 22 foods (49%) matched at level 0, and 4 foods (9%) matched at level 1. In the Dutch dataset, 3 foods (7%) matched at level 0, 6 foods (13%) matched at level 1, and 6 foods (13%) matched at level 2. In the UK dataset, 12 foods (27%) matched at level 0. Matches of level higher than 2 are not presented in the graph.

**Figure 6:**
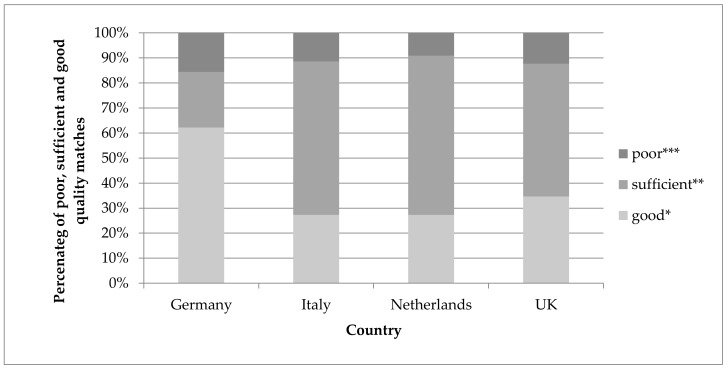
Percentage of poor, sufficient and good quality matches by country as selected and judged by the human experts. * Good—matches in all parameters; ** sufficient—matches in the name and group, but not in all descriptors; *** poor matches—all other matches.

The percentages of selected matches that were judged as good, sufficient and poor quality matches by the human experts within each country are shown in Figure 6. A match was judged as good when it matched in all attributes. A sufficient match was usually between foods matching in the English name and food group/subgroups but having different descriptors, or between a generic food from the food composition database and a more specific food from the food consumption dataset or vice versa. The remaining matches were judged as poor. The percentages refer to matching to the relevant national food composition database only, rather than using all available food composition databases. In Italy, The Netherlands and the UK the percentage of good matches ranged between 27 and 35%, with a mean value of 29.7% and standard deviation of 4.6% (Figure 6). For Germany, a higher percentage of good matches was reported (62%). The percentage of poor matches was rather similar across the countries and ranged between 9% and 16%, with a mean value of 12% and standard deviation of 2.9%.

**Figure 7:**
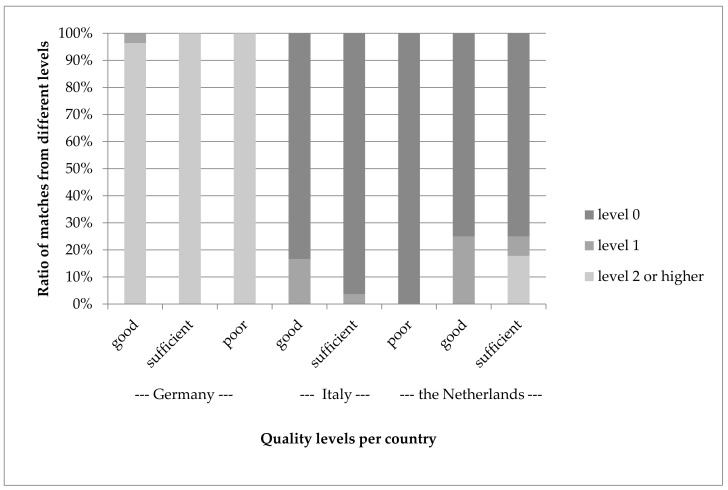
Distribution of quality levels such as indicated by the matching tool (level 0 = best quality) for foods with good, sufficient and poor match as judged by the human expert for three countries. For UK only level 0 matches were proposed so these are not included in the figure.

Figure 7 shows the relation between the quality level indicated by the matching tool and the quality of the match judged by the human experts for the selected matches.

**Figure 8:**
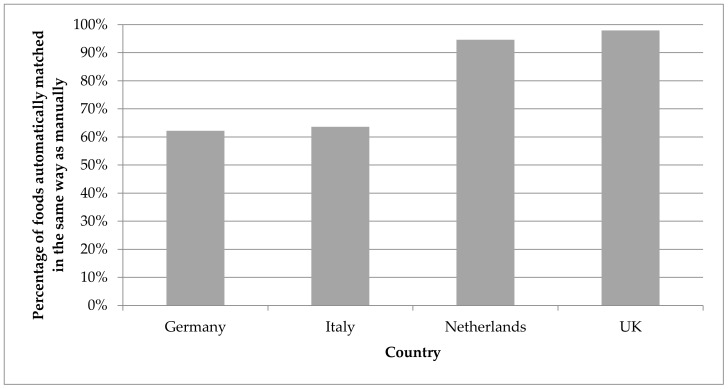
Percentage of foods from the food consumption survey that were assigned the same match from the food composition database in the testing exercises and in the previous manual match.

For the foods that were previously matched using a ‘manual’ procedure, 62% were matched with the same food from the food composition database in Germany, 64% in Italy and 95% in The Netherlands. For the UK, an experienced expert matched all but one food to the expected match (98%) (Figure 8).

**Figure 9:**
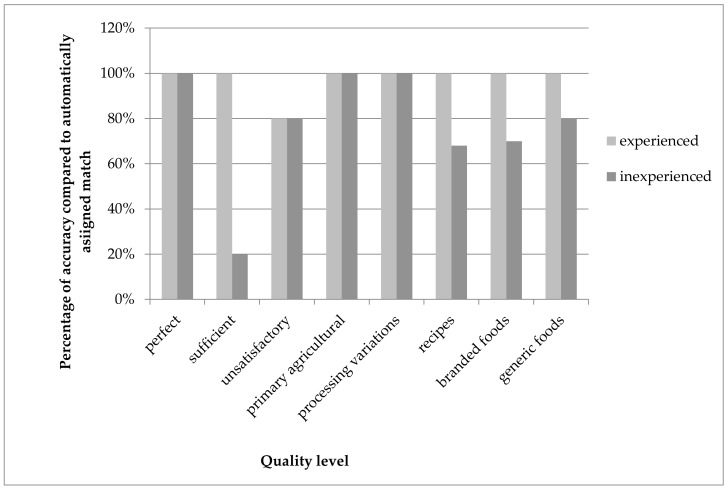
Percentage accuracy compared to the expected match for an experienced and an inexperienced human expert (results for UK).

The level of human expertise played an important role, at least in the training stage. Within the UK part of the validation, the effect of experience in working with food composition data could be studied. Figure 9 shows the accuracy of matching for the experienced and the inexperienced expert. The experienced expert (researcher) was able to retrieve an equal match (to the independent/expected match) on 48 of 49 occasions using the algorithm (for one food, no good match was available). For the inexperienced researcher, a lack of knowledge of the way foods can be described in the composition dataset, or of the products being tested, resulted in 10 foods not being matched equally. However, 38 of the 49 foods were still equal to the expected match.

The time complexity of the algorithm is not critical as the food matching algorithm outputs its results in an order of seconds. An experienced expert usually spends 10–15 min to find a good food match, while an inexperienced one may spend time twice as long.

## 5. Discussion

The case study for food matching between consumption and composition data was performed on diverse training and testing datasets from four countries. Some datasets, although extensive, were missing food information (such as food names, groups or descriptors), while others contained either too broad or mutually exclusive information unintentionally generated during the manual coding (e.g., the food name included the term “raw” and the descriptor “stir-fried”). This was welcomed because the aim of the case study was to identify gaps in data and food description by terms and facets that might influence food matching.

While the German and Dutch testing food consumption data were collected and described by GloboDiet and their food composition data were described by LanguaL, the matching of food consumption to composition data was performed by using the approach for matching GloboDiet to LanguaL (as described in Section 2.4.2). Dutch food composition testing data were fully described by English food names, food groups and subgroups, and LanguaL food descriptors. The German training set of food composition data was missing English names and food subgroups, and the LanguaL coded food items did not cover the variety of the extensive food consumption data. Although the German food composition dataset includes many food items (14,800), we could use compositional data and LanguaL codes for a limited subset of 1200 foods. Consequently, the German case study showed that querying by English names and groups/subgroups provided almost all matches at level 2 or higher. In many cases, however, when excluding food names and providing only the main food group without subgroups in the query, the results were much better (more level 0 and level 1 codes proposed). This can be explained by the fact that the provided training dataset only covered the main original GloboDiet food groups since the manual food matching in Germany the food subgroups were not relevant.

The Italian food consumption data collected by the INRAN-DIARIO 3.1 software were described by FoodEx2, and their food composition data were described by LanguaL. Also, the UK testing dataset was described by FoodEx2, and their food composition data were described by LanguaL. Therefore, the matching of Italian and UK food consumption to composition data was performed by using the approach for matching FoodEx to LanguaL (as described in Section 2.4.2). In the UK case, only level 0 matches were obtained, since only matching on food names was conducted. In Italy, no or few level 2 suggested matches were offered by the algorithm. This can be explained by the fact that the searched foods were associated with no or a few FoodEx2 descriptors, so also few unmatched aspects could occur. The average number of best quality matches (level 0) was 21 in Italy and over 12 in UK. These averages are high, and the offered best matches, therefore, were not very specific for the searched food, stressing the importance of the human expert to select the best match. Recently, we have improved the algorithm with more advanced matching by food names. In the version presented in this paper, simple matching of food names was applied as the focus was on food descriptors and facets as well as on groups and subgroups. Using machine learning and natural language processing approaches, food matching by names can become very efficient, as presented in Eftimov et al., 2017 [44].

Matches proposed by the algorithm for each country were subjectively validated by national experts. Different expectations and methods of judgements between the experts might explain some of the differences between countries and should be considered in the interpretation of the results. A poor match might be caused by certain foods or recipes not being available in the food composition database, or because the matching algorithm could not create the match. The high percentage of good matches in the German case study probably refers to the large number of foods in the German food composition table. A sufficient match was usually a match with the same food with different descriptors, or between foods that were more generic foods in the food composition database and more specific in the consumption survey data or vice versa. In the Dutch test, matches for fried or deep-fried foods were sufficient rather than good, because in the GloboDiet data these foods are the prepared foods, but excluding type of fat. Such types of foods are not available in the NEVO composition database (in contrast to the German BLS, which was adapted to the preparation methods of GloboDiet). Also, because information on brand names (available in the GloboDiet consumption data) could not be given as input facet for the matching, sufficient rather than good matches were often selected. The German experts found time consuming and difficult to judge matches because in many cases the number of options was too large (50 or in few cases even 150). 

It would be expected that good quality matches (as judged by the human expert) would be derived more often from the level 0 matches as suggested by the algorithm and that poor-quality matches would more often be suggested matches from a higher level. There was, however, no consistent relationship between both types of quality levels. The quality level, such as indicated by the algorithm, was mainly driven by differences between countries. For Germany, most suggested (and, therefore, selected) matches were only level 2 or higher as indicated by the matching algorithm.

The results indicate that a working knowledge of the UK composition dataset was important in increasing the accuracy of matches and returning the most relevant results. However, even for the inexperienced researcher, the ‘correct’ match (compared to the expected match) could still be achieved in most cases, although often more irrelevant matches were also returned. The exercise demonstrated the importance of knowledge of food descriptions when using the algorithm, particularly where only food names are used. The UK testing involved using the description straight from the subject food diary, which is seldom described as per the composition dataset. If facet codes are available, the accuracy of the food description is perhaps less important as the algorithm has another component to match on. Furthermore, it was observed in the Dutch testing that it was sometimes necessary to alter the search terms in order to find good matches. Since the involved experts knew the food composition and food consumption database very well, this resulted in a high percentage of equal matches, which might not have been obtained by inexperienced experts. Similarly, in the Italian testing, it was concluded that knowledge of the database to search in is crucial to increase the probability of exact matching. In the German testing, the expert did not change the food names in the search terms to improve the results. Also, a proper knowledge about the food composition database is necessary to classify the proposed matches as good, sufficient or poor. This became apparent during the testing of all food composition databases.

## 6. Conclusions

The case study showed that the most relevant requirement for efficient computer-based food matching is high quality of food consumption and composition databases. The type of classification and coding system used is of less importance. The quality of food information refers to the documentation of the data (including food description, component identification and descriptions of data source, sampling, analytical methods and laboratory performance), and food description is the important determinant for food matching. EuroFIR has produced a range of tools to help data compilers, including procedures for documenting data values, and has supported the development and publication of a standard for food data [13].

The main challenge in food matching is a diversity of datasets to be matched. They differ in the number of food items, the classification and coding system, the way of food naming, the level of details provided by descriptors, etc. A food matching algorithm needs to overcome these problems, considering all the information that is available. In the approach presented in this paper, we combined food information from different food composition and consumption datasets. For instance, food matching was enabled between a given food consumption dataset and all available food composition datasets. In this way, not only the training (learning) was more efficient, but also different countries could get an insight into the food information from other countries.

In the H2020 project RICHFIELDS [45], we are designing a new research platform aimed for collecting, harmonizing and linking food-, nutrition- and health-related data. In that project, we are further developing food matching methodology and technology. The collected information is also used to upgrade the existing Quisper ontology developed in the FP7 project QuaLiFY with the aim of harmonizing data from different information systems [46]. In this way, food matching will also be enabled with systems other than GloboDiet, LanguaL or FoodEx. In the present study, we matched foods considering only English names (besides description and classification terms) and this could be improved by considering also original (e.g., German) names.

## Figures and Tables

**Table 1:** Testing datasets of manually matched food consumption and food composition data.

Parameter	Germany	The Netherlands	Italy
**Survey name**	German National Nutrition Survey II (NVS II)	Dutch National Food Consumption Survey [35]	INRAN-SCAI
**period**	2005–2007	2007–2010	2005–2006
***n* of participants**	*n* = 13,926	*n* = 3819	*n* = 3323 (1300 households)
**age range**	14–80 years	7–69 years	0–97 years
**Dietary assessment method**	2 × 24 h-dietary recalls	2 × 24 h-dietary recalls	3 consecutive day food record
**administration**	Telephone	Telephone/Face to face home	Free-writing by the subject
**software**	GloboDiet Software (SW)	GloboDiet SW	INRAN-DIARIO 3.1 SW
**Food description**	The food description based on GloboDiet facets and descriptors, adapted to German-specific requirements	The food description based on GloboDiet facets and descriptors, adapted to Dutch-specific requirements	Predefined food categories + addition of new codes; FoodEx1 categories for the EFSA Comprehensive database assigned
**Data export format**	Microsoft Access database	Microsoft Excel	Microsoft Excel
**Data columns**	Food number, food name (English and German), food groups, facet and descriptor combinations, counts, food composition code, food composition name (German)	Food number, food name (English and Dutch), food groups, facet and descriptor combinations, counts, food composition code	Food number, food name, food group, food sub-group; food composition table (FCT) assigned code, FoodEx2 code and facets
**Data handling**	For the facet “brand name” only the information brand name known or not known was exported. Recipes are systematically split into their ingredients.	All brand name information is kept; recipes are systematically split into their ingredients	FoodEx2 mapping
**Number of unique combinations after data handling**	About 23,000 food and facet descriptor combinations	About 27,000 food and facet descriptor combinations	About 1500 food items (categories/products)
**Languages, in which food items are named**	German, English	Dutch, English	Italian, English

**Table 2:** Characteristics of the food composition databases, available in the EuroFIR information platform (except BLS) and used for training purposes. Since the study, presented in the paper, these characteristics have changed. Interested readers can find more details on the EuroFIR’s website [9].

Parameter	Germany	The Netherlands	Italy	UK
**Database name and version; year**	BLS (Bundeslebensmittelschlüssel) German nutrient database; version 3.01; 2013	NEVO Netherlands food composition database extended; version 3.0; 2011	INRAN Italian food composition database; 2008	Composition of Foods Integrated Dataset (CoFIDs); 2008
**Total number of foods**	~14,800 foods	2174 foods	790 foods	~3500 foods
**Number of foods used in the study**	~1200 foods	2174 foods	790 foods	~3500 foods
**Online database**	Database accessible through login at [36].	Database accessible through login at [37].	Database accessible through login at [38].	Database accessible through login at [39].
**Languages, in which food items are named**	German	Dutch, English	Italian, English	English
**Number of LanguaL coded foods**	1200 foods	2174 foods	790 foods	1682 foods

## Abbreviations

Cost99	Food and Agriculture COST Action 99
DAFNE	The Data Food Networking (DAFNE) initiative
DG-SANCO	European Commission Directorate-general for Health and Consumers Protection
EFSA	European Food Safety Agency
EPIC	European Prospective Investigation into Cancer and Nutrition
EuroDISH	EU-funded (FP7) project “Studying the need for food and health research infrastructures in Europe”
EuroFIR AISBL	The international, member-based, non-profit Association under Belgian law, set up in 2009 to ensure sustained advocacy for food information in Europe
FAO/INFOODS	The forum through which international harmonization and support for food composition activities can be achieved and advocated
FoodEx	EFSA’s food classification and coding system
FP7	Framework Programme 7—EU Research and Innovation programme
GloboDiet	The international standardized 24-h dietary recall methodology
H2020	Horizon 2020—EU Research and Innovation programme
LanguaL	The international framework for food description
RICHFIELDS	H2020 project “Research Infrastructure on Consumer Health and Food Intake for Escience with Linked Data Sharing”
QuaLiFY	EU-funded (FP7) project for personalized nutrition “Quantify Life—Feed Yourself”
